# Density of a cryptic Australian small mammal: The threatened Julia Creek dunnart (*Sminthopsis douglasi*)

**DOI:** 10.1002/ece3.11674

**Published:** 2024-07-02

**Authors:** Alice H. Bakker, Charlotte R. Patterson, Greg Mifsud, April E. Reside, Susan Fuller, Andrew M. Baker

**Affiliations:** ^1^ Faculty of Science, School of Biology and Environmental Science Queensland University of Technology Brisbane Queensland Australia; ^2^ Centre for Data Science Queensland University of Technology Brisbane Queensland Australia; ^3^ Greg Mifsud Consulting Toowoomba Queensland Australia; ^4^ School of the Environment University of Queensland Brisbane Queensland Australia; ^5^ Biodiversity and Geosciences Program, Queensland Museum South Brisbane Queensland Australia

**Keywords:** carnivorous marsupial, Dasyuridae, live trapping, mark–recapture, small mammal, threatened species

## Abstract

Globally, hundreds of mammal species face the threat of extinction in the coming decades, and in many cases, their ecology remains poorly understood. Fundamental ecological knowledge is crucial for effective conservation management of these species, but it is particularly lacking for small, cryptic mammals. The Julia Creek dunnart (*Sminthopsis douglasi*), a threatened, cryptic carnivorous marsupial that occurs in scattered populations in the central west of Queensland, Australia, was once so poorly studied that it was believed extinct. Sporadic research since its rediscovery in the early 1990s has revealed that *S. douglasi* is distributed across land at risk from many threats. Fundamental knowledge of *S. douglasi* population density is urgently required to inform conservation management at key sites, yet the species has historically proven hard to detect. Indeed, the status of the largest known population of *S. douglasi*, in Bladensburg National Park, is unknown. Here, we conducted a population study on *S. douglasi* at two sites within Bladensburg National Park via live mark–recapture surveys during 2022 and 2023. From likelihood‐based spatially explicit capture–recapture (SECR) modelling we provide the first estimates of density and population size for *S. douglasi*. Live trapping resulted in captures of 49 individual *S. douglasi* (with 83 captures total, including recaptures). We estimated *S. douglasi* to occur at a density of 0.38 individuals ha^−1^ (0.25–0.58) at one site and 0.16 individuals ha^−1^ (0.09–0.27) at another site, with an estimated mean population size in suitable habitat at Bladensburg National Park of 1211 individuals (776–1646). Our *S. douglasi* density estimates were similar to that reported for other threatened small mammals in Australia. We also found evidence of extreme *S. douglasi* population fluctuations over time at Bladensburg National Park, which is of concern for its future conservation. Our study has provided the first estimate of density for *S. douglasi*, a threatened dasyurid species from the Mitchell Grass Downs of central western Queensland, Australia. Our research provides crucial population data to assist the management of this poorly studied species. We demonstrate a method that can be applied to species with low detection probability to ultimately help address the mammal extinction crisis faced by Australia and the rest of the world.

## Graphical abstract

Here, we conducted a population study on the Julia Creek dunnart (*Sminthopsis douglasi*), a threatened, cryptic carnivorous marsupial that occurs in scattered populations in the central west of Queensland, Australia. Our study provides the first estimate of density at two sites within Bladensburg National Park via live mark–recapture surveys conducted during 2022 and 2023.This is crucial population data to assist in the management of this poorly studied species.

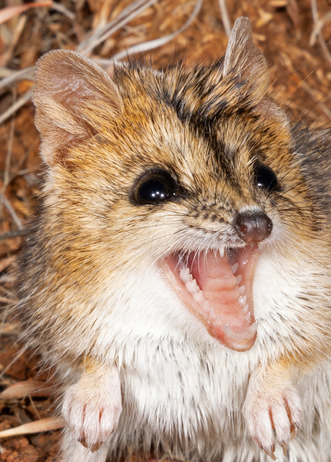

## 1 INTRODUCTION

It is now widely accepted that Earth is experiencing the sixth and first human‐induced, mass extinction event (Ceballos et al., 2010). Approximately 20% of all vertebrate species on Earth are threatened with extinction in the wild (Pereira et al., 2010), and it is estimated that one million species are at risk of extinction in the coming decades globally (Toussaint et al., 2021), including 558 mammal species by 2100 (Andermann et al., 2020) and seven mammal species in Australia by 2038 (Geyle et al., 2018).

Since European settlement in 1788, Australia's terrestrial mammal fauna has suffered severe declines and extinctions (Burbidge et al., 2009; Geyle et al., 2018). Leading causes of these declines include the arrival and spread of introduced predators such as feral domestic cats (*Felis catus*) and the European red fox (*Vulpes vulpes*), habitat loss, impacts by livestock and feral herbivores and diseases (Woinarski et al., 2015). Forty of Australia's mammal species have gone extinct since European settlement (about 40% of total global mammal extinctions; Burbidge, 2024) and 21% of the remaining species are classified as threatened (Cardillo & Bromham, 2008; Song et al., 2021; Woinarski et al., 2019). Additionally, evidence suggests that Australia's mammals within the weight range of 35 g–5.5 kg – a classification commonly known as the ‘critical weight range’ — are especially susceptible to extinction and have suffered significant reductions in their geographical distribution compared to their historical range (McKenzie et al., 2007; Moseby et al., 2009). It is a widely held view that species within this weight range have experienced the most severe declines (Johnson & Isaac, 2009).

Monitoring is fundamental to the management of threatened mammal species, so that population trends can be identified and baseline data established for a status assessment (Farhadinia et al., 2017). Such population monitoring efforts are urgent, as species responses to management strategies must be assessed and management adjusted accordingly before extinctions occur (Kinnear et al., 2017; Lunney et al., 2016; Wayne et al., 2015). Baseline surveys to establish the population status of threatened small mammals is particularly challenging for cryptic species, which might place them at greater risk of ineffective management.

The Julia Creek dunnart (*Sminthopsis douglasi*) is a cryptic, small mammal that requires long‐term monitoring to inform its management. *Sminthopsis douglasi* is an Australian carnivorous marsupial in the family Dasyuridae, and although it is the largest of the dunnarts, it falls well within the Critical Weight Range (maximum 70 g; Woolley, 1995). Originally described by Archer (1979), the species was thereafter presumed extinct until new records were found between 1991 and 1992 (Woolley, 1992). The distribution is presumed to be restricted to the Mitchell Grass (*Astrebla* spp.) Downs (Woolley, 1992) and Desert Uplands bioregions of central western Queensland (Queensland Government, 2009) where the species relies heavily on the grass‐covered deep cracks and tunnel networks in the black clay soil for shelter during the day (Mifsud, 1999). Nocturnal activity, underground habitat, presumed low abundance and susceptibility to a range of ongoing threats, make it a species in urgent need of monitoring. *Sminthopsis douglasi* is currently listed as Endangered in Queensland (Queensland Nature Conservation Act, 1992), Vulnerable under Australian federal legislation (EPBC Act, 1999) and Near Threatened on the IUCN Red List (IUCN, 2019). As of 2003, there were 25 locations where *S. douglasi* had been found across the Mitchell Grass Downs and Desert Uplands bioregions (Queensland Government, 2009). However, the occurrence of the species is presumed to be patchy and its abundance low (Kutt, 2003). This presumption is in part based on the fact that density of the species has never been estimated. Density estimates play a crucial role in guiding conservation efforts by providing essential information about the status, distribution and habitat requirements of species (Williams et al., 2002), enabling evidence‐based decision‐making aimed at ensuring the long‐term survival and recovery of vulnerable populations (Bradley et al., 2017).

In the last 25 years, records of *S. douglasi* have been sparse and monitoring efforts limited. No estimates of population density or population size have been made. Meanwhile, the landscape *S. douglasi* occupies (including two national parks [Mifsud, 1999; Woolley, 1992] but mostly private land [Smith et al., 2007]) and its associated threats (predation by ferals, habitat fragmentation and climate change [Kutt, 2003]) are likely to have worsened due to changes in land use and associated habitat loss, chiefly agriculture and grazing of cattle and sheep (Ward et al., 2021). Long‐term monitoring of *S. douglasi* populations is therefore needed to better understand the species’ response to such threats and to facilitate effective conservation management of wild populations.

The purpose of this study was to provide the first estimate of *S. douglasi* population density via a two‐year mark–recapture study in a key habitat, Bladensburg National Park and to build upon our knowledge of Julia Creek dunnart biology. Bladensburg National Park has been identified in the National Recovery Plan (Queensland Government, 2009) and via habitat modelling (Smith et al., 2007) as an important area for ongoing conservation management. The park is actively managed to eradicate introduced herbivores, feral predators and weeds such as prickly acacia (*Acacia nilotica*), that are the suspected leading causes of the species’ potential decline. There have been sporadic surveys for *S. douglasi* in Bladensburg National Park (Baker, 2013; Mifsud, 1999, 2000, 2001a; Rich, 2009, 2011 [unpublished]), which, although temporally patchy, provide records to broadly assess population trends (ranging from 0% to 6.75% *S. douglasi* capture success).

Based on historical records that indicate the species is at risk of decline throughout its range (Queensland Government, 2009), we hypothesised that capture success and subsequent density estimates of *S. douglasi* in the study area would be low (less than 1 individual ha^−1^). We also expected that the maximum movement between traps would be substantial (up to hundreds of metres), considering the predicted home range size of 0.25–7.13 hectares (Mifsud, 1999). Our research addressed several recommendations of the *S. douglasi* National Recovery Plan (Queensland Government, 2009), including conducting surveys to verify *S. douglasi* presence/absence in areas of suitable habitat, continuing monitoring of *S. douglasi* populations, implementing monitoring programs at significant sites to track abundance patterns and investigating interactions of *S. douglasi* and sympatric small mammal species.

## 2 METHODS

### 2.1 Study area

**FIGURE 1:**
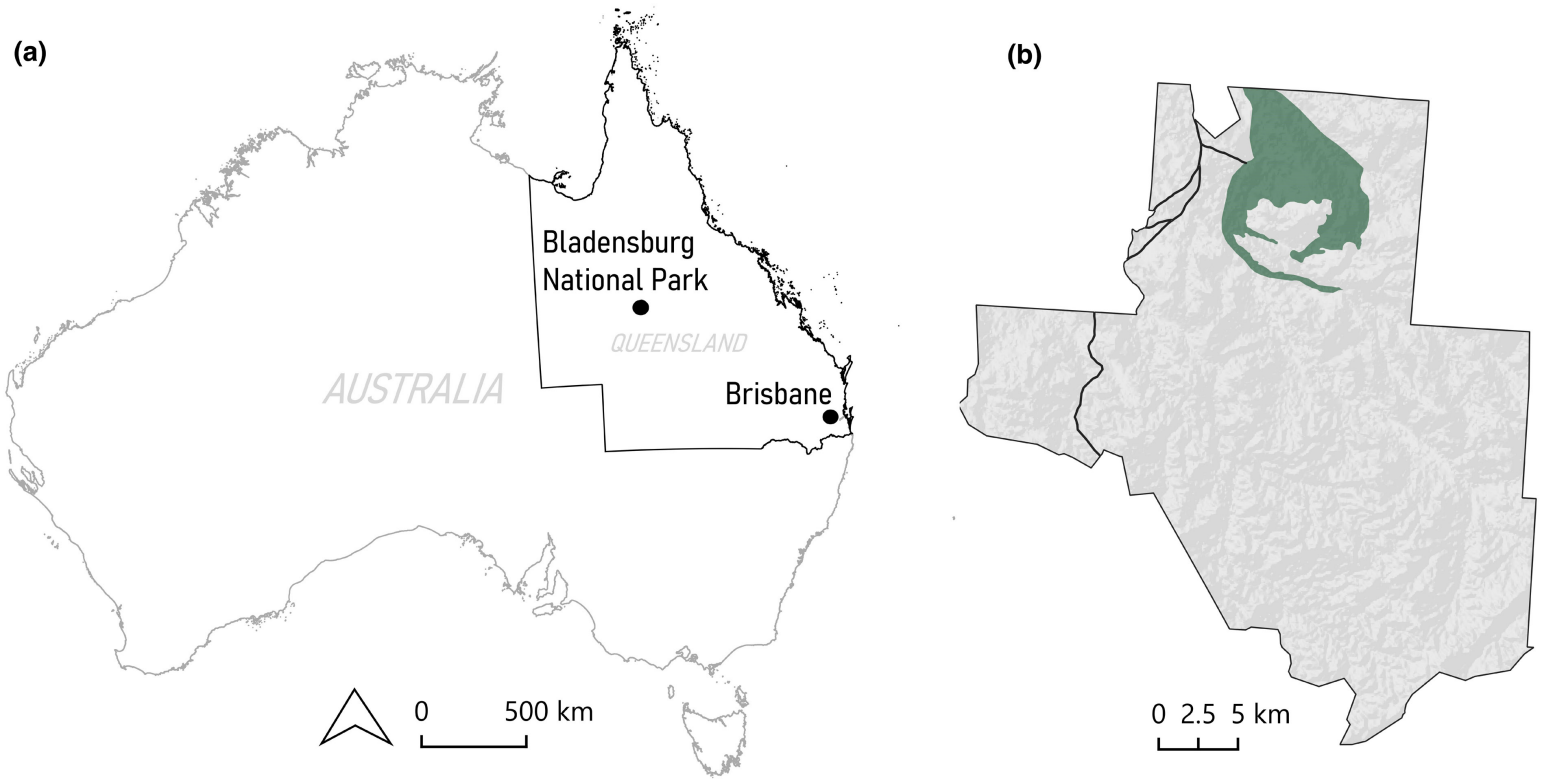
(a) The location of Bladensburg National Park, ~1147 km north‐west of Queensland's capital city, Brisbane. (b) Bladensburg National Park boundary, with the habitat mask (shaded green) in the northern section of the park. The shaded area, which contains the only appropriate habitat in the park to support *Sminthopsis douglasi*, was used to estimate the population size.

**FIGURE 2:**
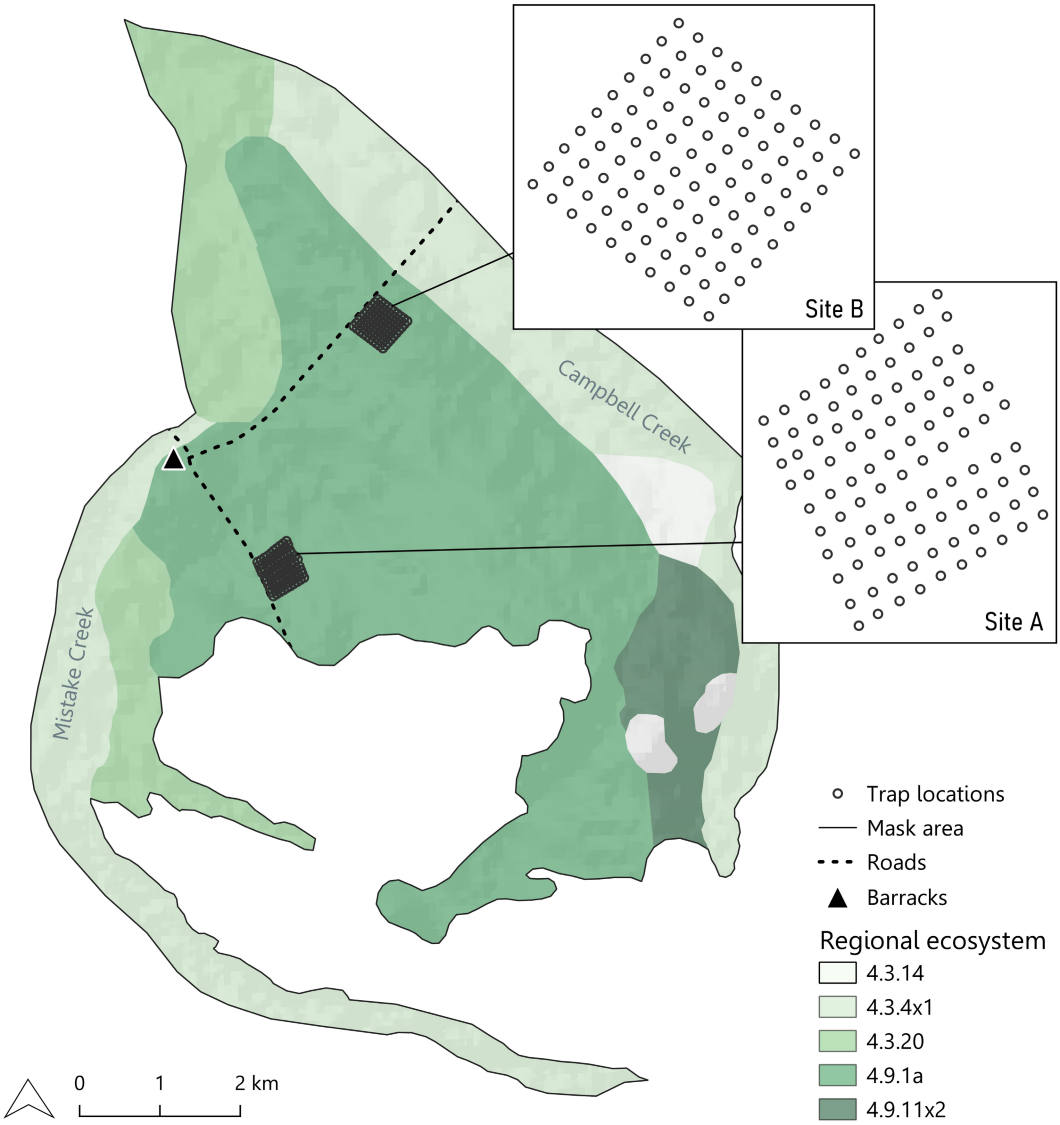
The location of study Sites A (Scrammy) and B (Campbell's) inside Bladensburg National Park with associated Regional Ecosystems, defined from the ‘Biodiversity status of 2021 remnant regional ecosystems – Queensland series v.13’ shapefile. The boundary displayed is a habitat mask of appropriate habitat for *Sminthopsis douglasi*, defined from the Regional Ecosystems. The habitat mask was used to estimate the population size of *S. douglasi* in the national park.

Our study was conducted at two sites in Bladensburg National Park, approximately 20 km south of Winton in Queensland, Australia (denoted as Site A and Site B; Figures 1 and 2). Bladensburg National Park occurs within the Mitchell Grass Downs bioregion and has an average annual rainfall of 634 mm (Bureau of Meteorology, 2023). Dominant plant species at the study sites include *Astrebla lappacea*, *A. pectinata*, *Aristida* sp., *Eragrostis* sp. and *Iseilema vaginiflorum* (Radford et al., 2001). Site A, ‘Scrammy grid’ (22°31.678′ S, 143°02.767′ E), was selected based on existing (sporadic) *S. douglasi* trapping effort and records (Mifsud, 2001a, 2001b; Figure 2). The second site, Site B, ‘Campbell's grid’ (22°30.103′ S, 143°03.510′ E), was chosen as a comparable trapping location on the basis that it occurs in the same Regional Ecosystem (RE4.9.1a; Queensland Government, 2023a; Queensland Government, 2023b) as Site A and is close to locations within the park where *S. douglasi* has historically been found (Figure 2; traps at Site B were minimum 2.6 km straight line distance from those at Site A). Regional Ecosystem 4.9.1a is defined as ‘*Astrebla lappacea* tussock grassland to closed tussock grassland, commonly with *Aristida latifolia* and *Iseilema vaginiflorum*. Emergent *Atalaya hemiglauca* and *Ventilago viminalis* may occur on undulating plains formed from Cretaceous mudstones’ (Queensland Government, 2021). Initially, we aimed to include a second location that also had historical capture records of *S. douglasi*. However, the cracking clay soils characteristic of the Mitchell Grass Downs region become quickly impassible after minimal rain (as little as 10 mm). Due to consistent wet weather in the early stages of the study (April 2022), access to this second location became impossible (being situated across an ephemeral creek), so Site B ‘Campbell's grid’ was chosen as a proximate alternative location (Figure 2). Based on reported average home range sizes (Mifsud, 1999; Woolley, 2016a), the two sites (A and B) were selected to be more than eight hectares (2.5 km straight line distance) apart, thus being independent and minimising the chance of trapping the same individuals at both sites.

### 2.2 Live trapping regime

All animal trapping and handling was conducted under the auspices of Queensland Department of Environment, Science and Innovation (DESI) Permit P‐PTUKI‐100171210 and QUT Research Ethics Permit 5154.

Mark–recapture live trapping was carried out between April 2022 and July 2023 across seven sessions and two sites. Trapping was conducted using Elliott‐style (type A) aluminium folding traps (as per Mifsud, 2001a). Each of the two sites consisted of 100 traps laid out in a 10 × 10 grid formation that was 500 × 500 m in extent. Each trap was set 50 m apart as per Mifsud (1999, 2001b) and a 50 m buffer was left between the dirt road and the start of the trapping grids to avoid potential road edge effects that may otherwise influence capture rate (Figure 2). Trap position was determined by GPS coordinates mapped via Google maps, with each transect walked by researchers who staked out thin, white 1.5 m‐long fibre glass marker posts with pink flagging tape, marking each trap location. Due to high summer temperatures and rainfall, which can cause unsafe fieldwork conditions (e.g., site inaccessibility, heat stress, exposure to snakes), trapping was planned primarily for autumn‐winter months with less extreme conditions. Previous studies indicated that *S. douglasi* can be detected in similar abundance throughout the year (Mifsud, 1999, 2000, 2001a, 2001b).

Each trap was baited with a mixture of peanut butter and bacon at an approximate ratio of 980 g of peanut butter to 250 g of bacon. The bait was rolled into a ball and wrapped in a square of biodegradable toilet paper to minimise mess in and around the traps and for time efficiency when baiting, following methods described by Mifsud (2001b). When traps were reset before sunset, the bait ball integrity was inspected for damage by ants. We replaced baits if they had dried out or if ants had eaten most of the ball. Despite ants accessing the bait in traps, there was no evidence of their interference with trapped mammals. Regardless of bait ball condition, rebaiting of all traps was conducted on the fourth day of trapping per session (Mifsud, 2001b).

The minimum trapping effort recommended to successfully survey for *S. douglasi* is seven consecutive nights (Mifsud, 2001b) and we aimed to match this in every trapping session. However, due to wet weather, which rapidly restricted access to the sites, seven consecutive trap nights at each site were not always achieved (see Table 1). When rain prevented access, traps were kept closed until the ground had dried sufficiently to continue trapping (typically between 1 and 4 days). Where possible, a total of 700 trap nights was conducted during each trapping session.

**TABLE 1:** Trapping summary of the mammal species captured at Site A (Scrammy) and Site B (Campbell's) in Bladensburg National Park in 2022 and 2023.

Date	Site	No. of trap nights	No. of captures of each species trapped (% trap success)		
			*Sminthopsis douglasi*	*Rattus villosissimus*	*Sminthopsis macroura*
21/04/2022	A	100^a^	0 (0)	0 (0)	0 (0)
31/05/2022–07/06/2022	A	700	2 (0.29)	1 (0.14)	0 (0)
02/06/2022–08/06/2022	B	600	3 (0.5)	0 (0)	0 (0)
11/07/2022–18/07/2022	B	700	0 (0)	0 (0)	0 (0)
13/07/2022–20/07/2022	A	700	12 (1.71)	0 (0)	0 (0)
23/08/2022–28/08/2022	A	500	2 (0.4)	0 (0)	0 (0)
25/08/2022–28/08/2022	B	300	0 (0)	0 (0)	0 (0)
13/04/2023–21/04/2023	A	700	4 (0.57)	25 (3.57)	1 (0.14)
13/04/2023–21/04/2023	B	700	0 (0)	0 (0)	0 (0)
28/05/2023–04/06/2023	A	700	28 (4)	19 (2.71)	0 (0)
28/05/2023–04/06/2023	B	700	15 (2.14)	0 (0)	0 (0)
14/07/2023–21/07/2023	A	700	8 (1.14)	35 (5)	0 (0)
14/07/2023–21/07/2023	B	698	9 (1.29)	1 (0.14)	0 (0)
Total		7798	83 (1.06)	81 (1.04)	1 (0.01)

*Note*: Captures and trap success for small mammal species in each trapping event is shown.

^a^ An unseasonal 150–200 mm inches of rain in 48 h prevented access to the site for 7 days, precluding further trapping on this trip.

Traps were set late in the afternoon, checked at dawn the following morning, then cleared and closed. Captured target species (dasyurids) were taken back to the national park barracks (~2–4 km distance) where we processed the animals at a secure and cool location. For dunnarts, measurements of the tail width, tail length, testis length (males), left and right pes (hindfoot) length, head‐body length and left ear length were taken with electronic Mitutoyo callipers, and body weight was measured with a Pesola spring balance. Three dunnart species are known to occur at Bladensburg National Park – *S. douglasi*, *S. macroura* and *S. crassicaudata* (Tighe, 2022). Thus, to ensure accurate identification, for each dunnart capture, photos were taken of the tail, top and bottom of hind feet, ventral and dorsal hair colouring, front and back of ear, testis (male) or pouch (female), teeth and face colouring. An ear clip (DNA), scats, cloacal swab and fur clip (odour samples) were collected for use in parallel studies. Reproductive condition was assessed and age (juvenile or adult) was determined by teeth assessment (i.e., state of third upper premolar teeth – presence of deciduous P^3^ or undescended adult P^3^ was deemed juvenile; fully descended adult P^3^ was deemed adult; Woolley, 2016b). A micro‐chip with a passive integrated transponder (PIT) tag (Microchips Australia – Trovan ID‐162B [1.4] ISO FDX‐B Midichip) was administered into the scruff of the neck for recapture identification. The dasyurids were held at the park barracks during the day until dusk, whereupon they were released at the point of capture. Individuals of non‐target (i.e., non‐dasyurid) species were identified and immediately released at point of capture.

### 2.3 Species identification

**FIGURE 3:**
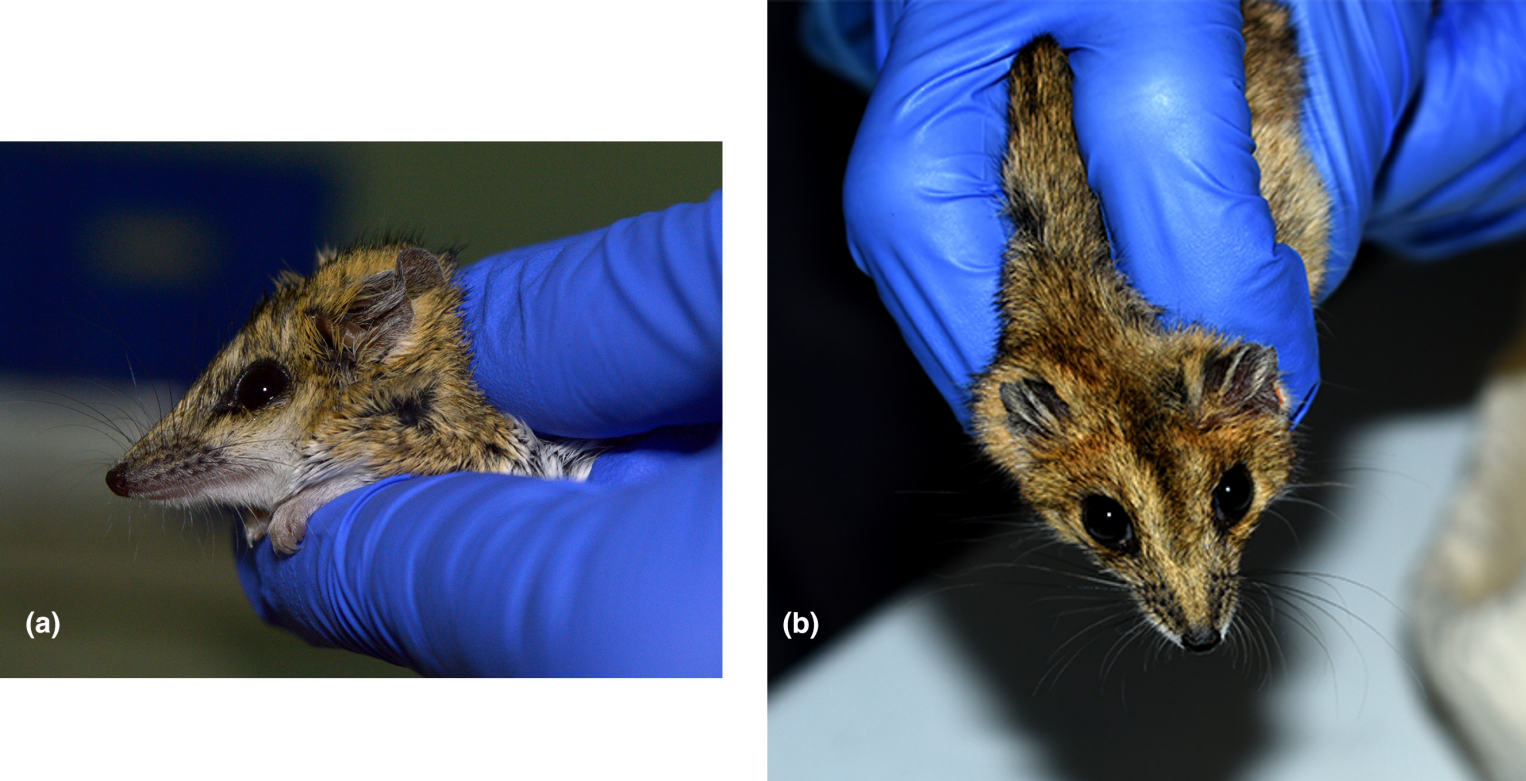
Morphological similarities between *Sminthopsis macroura* (a) and *S. douglasi* (b). Note the similarity in overall fur colour.

An Australian mammal field guide (Van Dyck et al., 2013) and expert elicitation (A.M. Baker, G. Mifsud, A. Bakker) was used to identify mammals to species. *Sminthopsis douglasi* was distinguished from the morphologically similar *S. macroura* primarily by a pes length measurement of >20 mm (in *S. douglasi*) in combination with weight and maturity (juvenile versus adult). For example, a *Sminthopsis* individual that was adult with a pes length of <20 mm and other corroborating features (body weight typically less than 26 g, fur colour, ear length, tail length, etc.) was deemed to be *S. macroura* or *S. crassicaudata*, whereas a juvenile individual with a pes length close to or >20 mm, or an adult with a pes of >20 mm and other corroborating features (body weight typically more than 25 g, fur colour, ear length, dark hairs around eye and on tail tip, etc.) was identified as *S. douglasi* (as per Archer, 1979; Mifsud, 1999; Van Dyck et al., 2013; Woolley, 1992, 1995; Figure 3). *Sminthopsis macroura* was distinguished from *S. crassicaudata* by a combination of relative head‐body: tail length, fur colour, head features (shape of head, relative eye and ear size) and body weight (Van Dyck et al., 2013).

**FIGURE 4:**
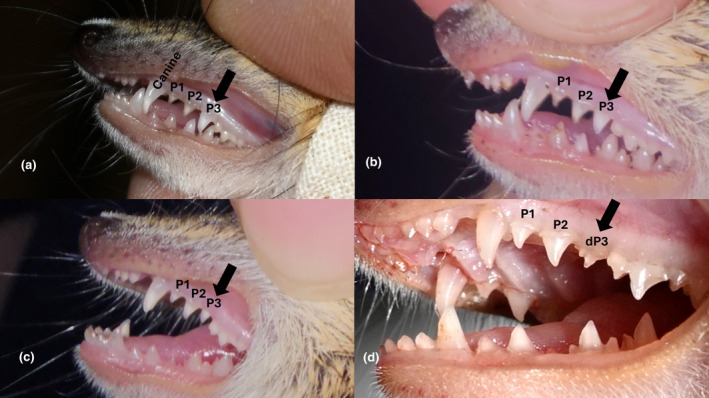
Examples of ageing dasyurids via teeth assessment. The set of three upper premolars are positioned immediately behind the enlarged (curved) canine tooth (as labelled). Black arrows indicate the third upper premolar tooth in each case: (a) adult, with third premolar bigger than the second; (b) juvenile, with 3rd premolar about the same size as the second; (c) juvenile, with 3rd premolar smaller than the 2nd; and (d) juvenile with deciduous third premolar (dP^3^, milk tooth). Note the difference in deciduous P^3^ and adult P^3^ morphology. In the latter case (d), the third adult premolar has not yet begun to descend, which happens as the deciduous P^3^ is lost.

**FIGURE 5:**
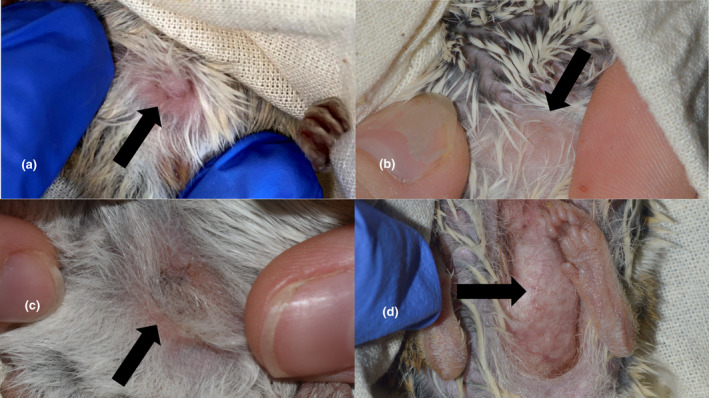
Examples of pouch conditions in *Sminthopsis*. (a) is a juvenile with an immature pouch score; (b, c) are adults with immature pouch scores; and (d) is an adult with a lactating pouch score. The black arrows indicate the pouch area.

**FIGURE A1:**
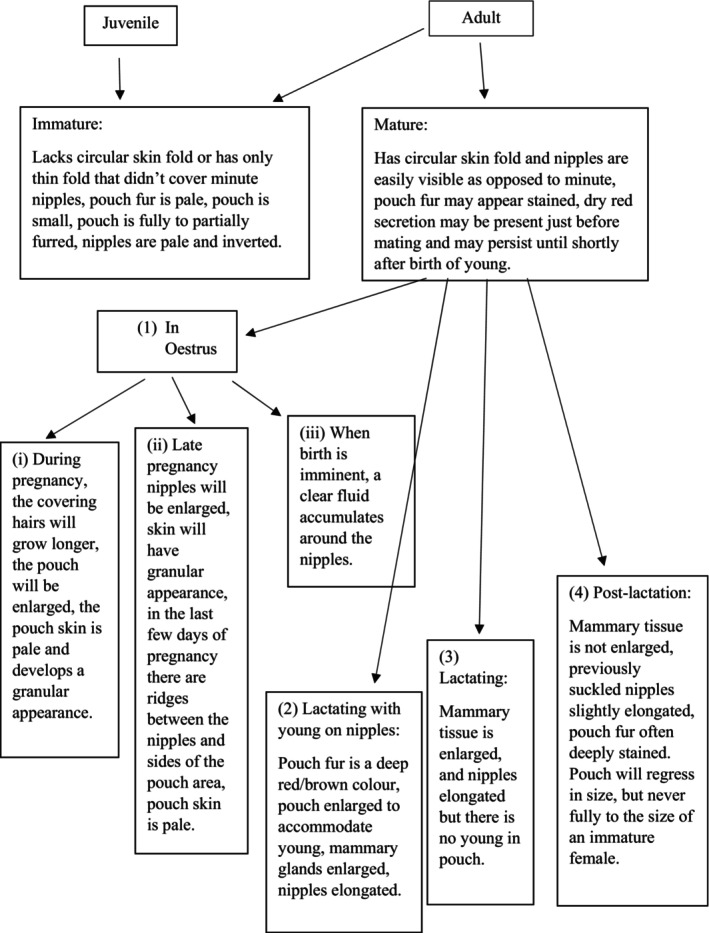
Pouch condition score of *Sminthopsis* spp. assessment criteria.

Maturity of dasyurid species was determined via teeth examination to assess crown height and condition of premolar teeth. If the third upper premolar tooth was larger (in crown height) than the second upper premolar, it was classed as an adult; if the third upper premolar tooth was smaller than or the same size as the second upper premolar, or if the milk tooth (deciduous P^3^) was still present, it was classed as a juvenile (Woolley, 2016b; Figure 4). The third premolar of adult *S. douglasi* descends between 130 and 170 days, but females can reach sexual maturity as early as 119 days, depending on available food resources. This means that females (but typically not males, which mature a month or more later) may be capable of breeding before the third adult upper premolar fully descends (Woolley, 2016b). Therefore, relying solely on the state of the third upper premolar tooth is not a definitive method for judging maturity in females. However, since breeding usually begins in October each year, the status of the third upper premolar tooth serves as a convenient marker for age category delineation of females when trapping between April and September, because mating should not occur later than February/March. To assess the maturity of females in more detail and assist with identification, a pouch condition score was also assigned as per Woolley (1966) (Figures 5 and A1).

### 2.4 Spatially explicit capture–recapture analysis

Estimating population density via spatially explicit capture–recapture (SECR) modelling is a robust approach that, unlike traditional mark–recapture methods, utilises spatial information about individual animal capture and recapture locations to estimate species detection probability (Efford, 2023). SECR models require repeat detections of multiple individuals to obtain a robust estimate of population density (Efford et al., 2009), so a substantial survey effort and fair recapture success is required, which can be challenging for cryptic or rare species.

Density of *S. douglasi* in the study area was estimated via likelihood‐based SECR (Borchers & Efford, 2008) using the *secr* package version 4.6.4 (Efford, 2023) for R version 4.3.1 (R Core Team, 2023). SECR models require a defined analysis region, which must be large enough to encompass the activity centres of all individuals within a population (Royle & Young, 2008). Our analysis region was a 300 m buffer around the traps, with the buffer width selected using the ‘*esa. plot*’ and ‘*RPSV*’ functions in *secr*. We compared simple models with exponential and half‐normal detection functions via Akaike's corrected information criterion (AICc) (Hurvich & Tsai, 1989) to select an appropriate detection function for *S. douglasi*.

Data from Sites A and B were included in a multi‐session model, with each trapping period at each site treated as a separate session. We therefore assumed that there was no movement of individuals between sites and that each sites’ population was closed between trapping sessions (i.e., no births, deaths, immigration or emigration; Otis et al., 1978). The assumption of site independence was supported by no recaptures between sites. We fitted seven candidate models and compared them via AICc. The first was a null model, with constant density and detection parameters among sessions. Due to observed variation in capture rates between Site A and Site B and among trapping months, we tested candidate models capturing effects of categorical predictors Site (A or B) and Season (Winter – June, July, August or Autumn – April, May). Among these candidate models, Site or Season influenced density, g0 (the one‐night probability of capturing an individual in a trap at the centre of its home range), or both density and g0, while other parameters were held constant. The most supported model (lowest AICc value) was selected for our final density estimate, with a ΔAICc of two and a moderate weight as the criteria for strong model support (Burnham & Anderson, 2002).

We used the final model to estimate population size of *S. douglasi* within Bladensburg National Park with the ‘*region.N*’ function of *secr* (Efford & Fewster, 2013). The national park contains ecosystem types that are known to be unsuitable for *S. douglasi*, so before estimating population size we delineated areas of likely *S. douglasi* habitat using prior knowledge of *S. douglasi* distribution (Augusteyn et al., 2018; Baker, 2013; Kutt, 2003; Lundie‐Jenkins & Payne, 2000; Mifsud, 1999, 2001a, 2001b; Queensland Government, 2022; Rich, 2011 [unpublished]; Smith et al., 2007; Woolley, 1992,  2000) within Regional Ecosystems of Queensland (‘Biodiversity status of 2021 remnant Regional Ecosystems – Queensland series v. 13’ shapefile, QLD Spatial Catalogue). Since our final model generated separate density estimates for Sites A and B, we combined inference from both sites by estimating population size twice across the entire habitat region from density estimates at Sites A and B, then took the average and standard error of the two estimates using the inverse‐variance method (Borenstein et al., 2009).

### 2.5 Live trapping comparison with previous studies

We compared live trapping effort and trap success using Elliott‐style traps for *S. douglasi* at Bladensburg National Park (site A) from the years 2000 to 2023 (see Table A1: Appendix B).

## 3 RESULTS

### 3.1 Live trapping

From the 7798 trap nights conducted across 2022 and 2023, we captured 49 *S. douglasi* individuals, with 83 total captures and 34 recaptures (Tables 1 and 2), representing an average capture success of 0.88% and 0.63 individuals/100 trap‐nights. This equated to one *S. douglasi* capture for every 159 trap nights. Site A had a higher average trapping success for *S. douglasi* of 1.12% compared to Site B, 0.66%. Overall, more males were caught than females (30 M, 19 F) and more adults than juveniles (35 adults, 14 juveniles; Table 2). Twelve female *S. douglasi* were recorded as juveniles with a pouch score of ‘immature’; eight were recorded as adult with a pouch score of ‘immature’; one was recorded as adult with a pouch score of ‘lactating’ (in April 2023) and three were recorded as adult with a pouch score of ‘post lactation’ (in July 2022 and July 2023). One individual was recorded as juvenile with a pouch score of ‘immature’ upon first capture on 1 June 2023, which then developed into an adult with a pouch score of ‘immature’ in a subsequent recapture on 15 July 2023. Highest trapping success for both sites occurred between the 28th of May to the 4th of June in 2023, where Site A trap success was 4% and Site B was 2.14%. Other species caught over the two‐year trapping period included one *S. macroura* and 81 *Rattus villosissimus* captures (*R. villosissimus* were not identified to individual; Table 1). Most *S. douglasi* individuals showed median trap‐revealed movements >50 m (equivalent to the trap spacing), which suggested that even individuals with home range centres outside of the trapping grid would have encountered traps (Efford, 2023).

**TABLE 2:** Results of trapping for *Sminthopsis douglasi* at Site A (Scrammy) and Site B (Campbell's) in Bladensburg National Park in 2022 and 2023.

Date	Site	No. of trap nights	No. captures	No. recaptures	Sex ratio M:F	Age ratio A:J	% trapping success
21/04/2022	A	100^a^	0	0	0:0	0:0	0
31/05/2022–07/06/2022	A	700	2	0	2:0	1:1	0.29
02/06/2022–08/06/2022	B	600	3	0	1:2	1:2	0.5
11/07/2022–18/07/2022	B	700	0	0	0:0	0:0	0
13/07/2022–20/07/2022	A	700	12	5	2:1	9:0	1.71
23/08/2022–28/08/2022	A	500	2	2	1:0	1:0	0.4
25/08/2022–28/08/2022	B	300	0	0	0:0	0:0	0
13/04/2023–21/04/2023	A	700	4	0	1:1	3:1	0.57
13/04/2023–21/04/2023	B	700	0	0	0:0	0:0	0
28/05/2023–04/06/2023	A	700	28	10	13:6	12:7	4
28/05/2023–04/06/2023	B	700	15	6	2:1	7:2	2.14
14/07/2023–21/07/2023	A	700	8	6	3:2	4:1	1.14
14/07/2023–21/07/2023	B	698	9	5	2:1	6:0	1.29
Total		7798	83	34	27:14	3:1	1.06

*Note*: ‘No. of individuals’ are total captures in a trapping session. ‘No. of recaptures’ is for each individual trapping session. Sex and age ratios in each trapping event are shown.

^a^ An unseasonal 150–200 mm inches of rain in 48 hours prevented access to the site for 7 days, precluding further trapping on this trip.

### 3.2 Density and population size

**FIGURE 6:**
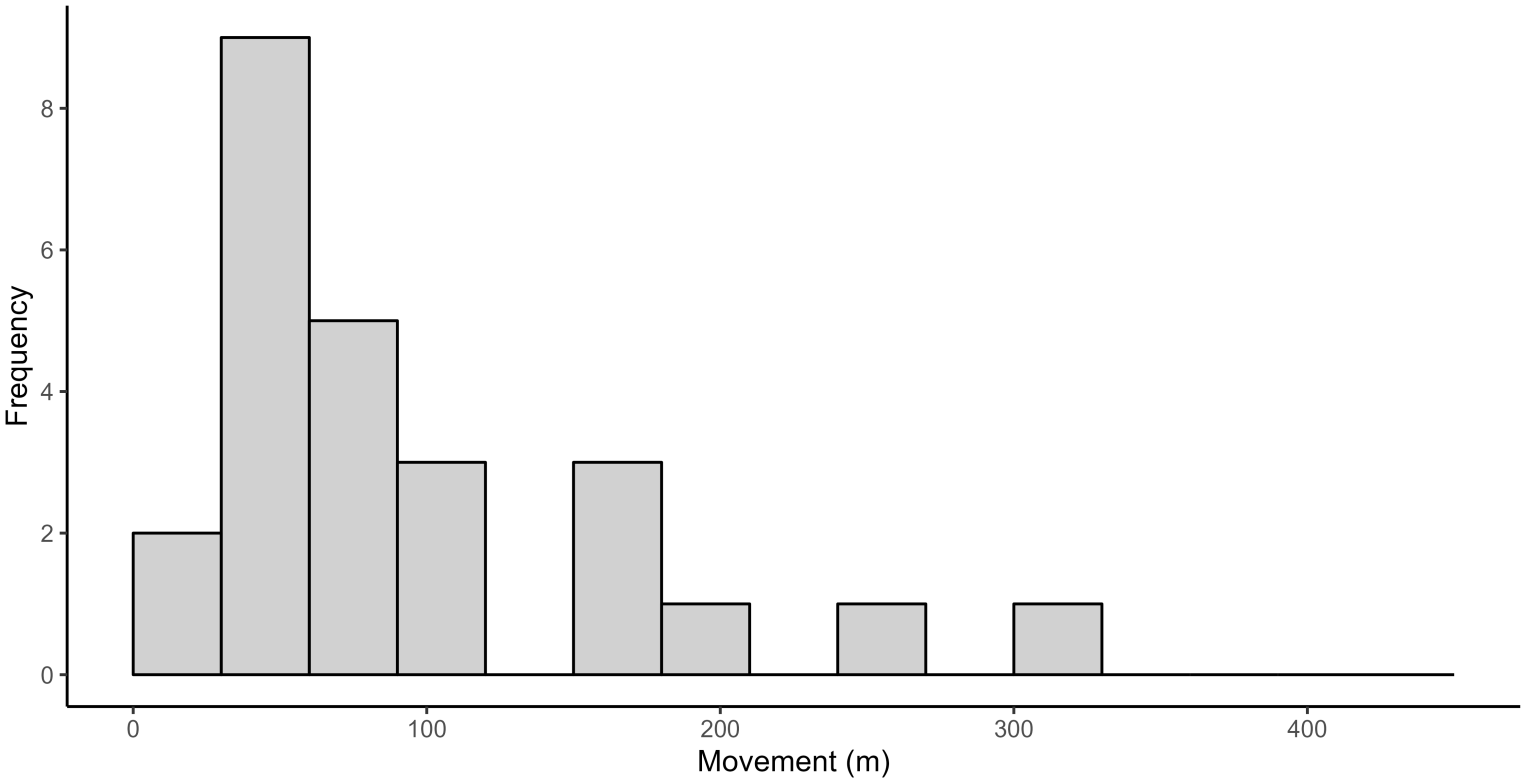
Frequency of *Sminthopsis douglasi* individuals in relation to the distance they moved (in metres) between consecutive captures.

After comparing models with the exponential and half‐normal detection functions via AICc, we selected the exponential function for subsequent model fitting. Comparison of models with and without Site and Season effects revealed the greatest support for a model where density varied by site, but detection parameters were shared across the sites (Table 3). Final *S. douglasi* density estimates from this model were 0.38 individuals ha^−1^ (95% CI 0.25–0.58) at Site A and 0.16 individuals ha^−1^ (95% CI 0.09–0.27) at Site B, while trapping parameters g0 and *𝜎* were 0.053 and 36.45, respectively. Our estimated mean population size of *S. douglasi* within areas of suitable habitat in Bladensburg National Park (5725 ha) was 1211 individuals (95% CI 776–1646). By summarising and plotting recapture locations, we determined that the median distance between individual recaptures was 61.19 m (range 47.38–320.85 m; Figure 6).

**TABLE 3:** Results of the AICc analysis of candidate models.

Model	Parameters	$\hat{D}$	95% CI	$\hat{g_{0}}$	$\hat{\sigma }$	Log likelihood	ΔAICc	AICc weight
Site.D *Site A*	*D*~Site *g*_0_ ~ 1 *σ* ~ 1	0.38	0.25–0.58	0.053	36.45	−489.86	0.00	0.65
Site.D *Site B*	*D*~Site *g*_0_ ~ 1 *σ* ~ 1	0.16	0.09–0.27	0.053	36.45	−489.86	0.00	0.65
Site.D.g0 *Site A*	*D*~Site *g*_0_ ~ Site *σ* ~ 1	0.40	0.25–0.64	0.049	36.44	−489.66	2.00	0.24
Site.D.g0 *Site B*	*D*~Site *g*_0_ ~ Site *σ* ~ 1	0.14	0.08–0.27	0.063	36.44	−489.66	2.00	0.24
Season.D *Autumn*	*D*~Season *g*_0_ ~ 1 *σ* ~ 1	0.33	0.22–0.51	0.054	36.45	−492.68	5.66	0.04
Season.D *Winter*	*D*~Season *g*_0_ ~ 1 *σ* ~ 1	0.19	0.12–0.32	0.054	36.45	−492.68	5.66	0.04
Site.g0 *Site A*	*D*~1 *g*_0_ ~ Site *σ* ~ 1	0.30	0.20–0.45	0.033	36.62	−492.91	6.10	0.03
Site.g0 *Site B*	*D*~1 *g*_0_ ~ Site *σ* ~ 1	0.30	0.20–0.45	0.059	36.62	−492.91	6.10	0.03
Season.g0 *Autumn*	*D*~1 *g*_0_ ~ Season *σ* ~ 1	0.28	0.19–0.41	0.062	36.63	−493.45	7.20	0.02
Season.g0 *Winter*	*D*~1 *g*_0_ ~ Season *σ* ~ 1	0.28	0.19–0.41	0.039	36.63	−493.45	7.20	0.02
Null	*D*~1 *g*_0_ ~ 1 *σ* ~ 1	0.26	0.18–0.39	0.054	36.46	−494.82	7.61	0.01
Season.D.g0 *Autumn*	*D*~Season *g*_0_ ~ Season *σ* ~ 1	0.33	0.20–0.52	0.056	36.48	−492.66	8.00	0.01
Season.D.g0 *Winter*	*D*~Season *g*_0_ ~ Season *σ* ~ 1	0.20	0.11–0.37	0.051	36.48	−492.66	8.00	0.01

*Note*: Models with Site or Season depicted an effect of site or season on density or trapping probabilility (*g*_0_). ~1 indicates a constant. The most well supported model (shaded) was Site.D. No other models were considered well‐supported. $\hat{D}$ is estimated density per ha and its 95% confidence interval, ${\hat{g}}_{0}$ and $\hat{\sigma }$ jointly define the estimated detection function, AICc weight is the model weight. Site‐ or season‐specific density or *g*_0_ values are presented and labelled under Model names.

### 3.3 Live trapping comparison with previous studies

Our historical comparison revealed that survey effort and trap success (Bladensburg NP, Site A) have varied over time, with the highest trapping effort in 2013 yielding zero *S. douglasi* captures (Baker, 2013) and the highest trapping success of 6.75% from 800 trap nights occurring in 2001 (Mifsud, 2001a). Capture rates in 2000–2001 (mean 5.0% *S. douglasi* trap success) were higher than in 2009–2013 (no captures) and notably higher than those in 2022–2023 (mean 1.2%; see Table A1: Appendix B for more details). These data indicate greater than tenfold fluctuations in *S. douglasi* captures per unit effort at this site over time.

## 4 DISCUSSION

Using mark–recapture data obtained from multiple live trapping surveys, our study has provided the first estimate of density for *S. douglasi*, a threatened dasyurid species from the Mitchell Grass Downs of central western Queensland, Australia. Based on generally low and declining captures in historical data, *S. douglasi* trap success and estimated density in the study area was expected to be low. Trapping in our study during 2022–2023 revealed a low density of 0.38 individuals ha^−1^ at Site A and 0.16 individuals at Site B, along with a predicted population size of 1211 *S. douglasi* in the Bladensburg National Park study area. Our population size estimate is based on two trapping grids and is extrapolated across likely areas inhabited by the species in the park, so it must be interpreted with caution. However, the density and population size estimates provide important insights into present population status of *S. douglasi* within its single largest area of protected habitat. Bladensburg National Park is likely to be the most significant population refuge for *S. douglasi* given that most of its predicted range is under private landholder use for cropping and stock rotation and is subject to a range of threats (Kutt, 2003).

Our *S. douglasi* density estimates (0.38 individuals ha^−1^, 0.16 individuals ha^−1^) are similar to estimates for other nationally threatened Australian marsupials, including the dasyurid crest‐tailed mulgara (*Dasycercus cristicauda*; 0.23 individuals ha^−1^; Thompson & Thompson, 2008), long‐nosed potoroo (*Potorous tridactylus*; 0.23–0.26 individuals ha^−1^; Mason, 1998) and numbat (*Myrmecobius fasciatus*; 0.01 individuals ha^−1^; Vieira et al., 2007). However, it is worth noting that some threatened Australian small mammals have higher densities (e.g., *Perameles gunnii* [Mallick et al., 2000] and *Pseudomys novaehollandiae* [Wilson et al., 2018]) and although Australian arid zone small marsupials typically have low densities (<1 individual ha^−1^), this may increase to up to 5 individuals ha^−1^ when conditions are favourable (Dickman et al., 2001). As expected, the densities of Australia's threatened small mammals typically are lower than those of other small mammals that are not listed, such as the dasyurid little red kaluta (*Dasykaluta rosamondae*) at 1.88 individuals ha^−1^ and the sandy inland mouse (*Pseudomys hermannsburgensi*s) at 4.90 individuals ha^−1^ (Thompson & Thompson, 2008).

From a global perspective, our density estimates for *S. douglasi* were substantially lower than density estimates of international threatened small mammals (albeit rodents), such as the salt marsh harvest mouse (*Reithrodontomys raviventris*; minimum density estimate 9.13 ± 2.70 individuals ha^−1^; Freeman et al., 2022), the Key Largo woodrat (*Neotoma floridana smalli*; minimum density estimate 3.1 individuals ha^−1^), the cotton mouse (*Peromyscus gossypinus allapaticola*; minimum density 15.5 individuals ha^−1^; Humphrey, 1988) and the Kondana soft‐furred rat (*Millardia kondana*; minimum density 2.01 individuals ha^−1^; Bajaru & Manakadan, 2020). However, Australian semi‐arid and arid small mammals are known to be highly mobile and occur at low densities, likely due to spatiotemporally patchy resources and low habitat productivity, so this may in part account for the generally lower small mammal densities found here (Dickman et al., 1995).

Based on our live capture data, the small mammal communities at the two sites in Bladensburg National Park consisted of two dominant species, *S. douglasi* and *Rattus villosissimus*, with a single *S. macroura* captured. However, several other rodent species (e.g., *Leggadina forresti* and *Pseudomys desertor*), as well as various dasyurids, such as *Planigale* sp. and *S. crassicaudata*, are known to inhabit the park (Tighe, 2022). *Sminthopsis douglasi* had a higher density at Site A than Site B. The differences in trap success at the two sites may relate to variable microhabitat resources, such as food availability, soil crack abundance, vegetation cover and distance to the nearby ephemeral creek bed. Examination of such habitat factors and how they might influence *S. douglasi* population dynamics across a range of suitable areas would be a useful avenue of future research.

We found the ratio of juvenile to adult *S. douglasi* remained consistently low throughout much of 2022 and 2023. However, in May 2023, there was a notable surge in juvenile numbers, accounting for nearly half the trapped population. This increase aligns with the documented captive breeding season of *S. douglasi* from June to February (Woolley, 2016b), although in the field, females were found to give birth from September until mid‐February (Mifsud, 1999). Given that females have been observed to rear up to two litters of young per breeding season in conditions where resources are abundant, juvenile recruitment can commence midway during the breeding season, from December onwards (Mifsud, 1999). Peak numbers of *S. douglasi* captures were in July 2022 and May–June 2023, indicating successful recruitment of independent offspring in the trap sites from the previous breeding periods. Thus, we expected high activity associated with the breeding season during the July 2022 trapping session (trap success 2.14%). The following year, juveniles were still being captured in July of 2023, suggesting late breeding events occurred (perhaps in February 2023) and the increased population numbers in the May–June 2023 trapping session (trap success 4%) may reflect the presence of these late‐bred juveniles prior to or during dispersal. Females in ‘post lactation’ condition were caught only in July in both 2022 and 2023. We caught one adult male individual between years (Site A) suggesting a potential life span of at least 11 months. No other individuals were captured across years, suggesting strong annual recruitment between 2022 and 2023 (Mifsud, 1999).

Our observed maximum movement of an *S. douglasi* individual between trap locations (321 m) was substantial, but the mean across all animals (61 m) was lower than expected, considering their predicted home range size of 0.25–7.13 hectares (Mifsud, 1999). We captured no individuals that had moved between trap sites, suggesting that the two sites were independent, and the limited predicted average movement of individuals within each site reinforces the likely independence of our two sites.

Some individuals exhibited ‘trap‐happy’ behaviour (Free & Leung, 2007; Harkins et al., 2019; Morris et al., 2011), and there were many recaptures. Indeed, there were some instances where we trapped an individual multiple times in one trapping session and on consecutive days. Such behaviour is not unusual in small mammal studies (Flowerdew et al., 2004) and has been recorded before in *S. douglasi* (Mifsud, 1999, 2001a, 2001b). Our results reinforce the general utility of Elliott‐style trapping for monitoring *S. douglasi*, which is notable, given many *Sminthopsis* are not readily captured using this method (Baker & Dickman, 2018).

The maximum trap success recorded in our study (4%) was lower than previous studies at Bladensburg National Park (Site A), where maximum trap success was 6.75% (Mifsud, 2001a, 2001b). Mifsud (2001a, 2001b) caught 92 individual *S. douglasi* with 171 total captures across 2100 trap nights, which is markedly more than the 49 individual individuals and 83 total captures from 7798 trap nights found in our study. However, considering all trapping conducted at Bladensburg National Park, trapping success has varied from 0% (Baker, 2013) to 6.75% (Mifsud, 2001a, 2001b). This suggests that the population abundance of *S. douglasi* at Bladensburg National Park fluctuates dramatically (more than tenfold), and this may be due to changes in rainfall, vegetation cover, food availability and formation of cracks for refuge (Mifsud, 1999). Fluctuating small mammal populations are not unusual in Australia (Moseby et al., 2023; Predavec & Dickman, 1994; Smith, 2015). However, if exacerbated by threats (e.g., climate change and feral predators), such dramatic fluctuations may be of conservation concern. Thus, an assessment of the environmental factors that may influence persistence and population dynamics of *S. douglasi* at Bladensburg National Park is required. However, at present, any formal analysis of population trends is precluded by the extremely patchy temporal monitoring that has been undertaken on the population at Bladensburg National Park over the last 25 years. We recommend that a long‐term program of annual monitoring be undertaken, particularly as the climate changes through El Nino‐La Nina oscillations (Wang & Hendon, 2007), which may be an important driver of mammal density across time.

In summary, our study has provided important density and population size estimates that will contribute to the conservation management of *S. douglasi*. No regular population monitoring has been conducted on this species for almost 25 years (Mifsud, 2001a). We suggest that the density of the species is low, but the total population of *S. douglasi* at Bladensburg National Park is currently abundant relative to previous studies, which provide evidence of extreme fluctuation over time. Further annual monitoring at this location is required and at other populations, such as the other protected area (Moorrinya National Park), but also on privately owned (cattle‐grazed) areas, to elucidate the status and persistence of the species in areas subject to land‐use change and other threats. Research should also be conducted to determine why trapping success varied at the two study sites investigated in our study, as it suggests within‐ecosystem variation in habitat preference.

Our research has shown that important baseline population data fundamental for conservation can be retrieved even for cryptic, small mammal species that are likely to occur patchily in both space and time. Such remote work is logistically challenging and expensive, but it will be crucial if Australia is to learn important conservation lessons from its recent past, to prevent further mammal extinctions and ultimately inform management strategies that can be applied on both the national and global stage.

## AUTHOR CONTRIBUTIONS

**Alice H. Bakker:** Conceptualization (equal); data curation (lead); formal analysis (equal); investigation (equal); methodology (equal); writing – original draft (lead); writing – review and editing (lead). **Charlotte R. Patterson:** Formal analysis (lead); writing – review and editing (equal). **Greg Mifsud:** Conceptualization (equal); methodology (equal); writing – review and editing (equal). **April E. Reside:** Conceptualization (equal); investigation (equal); methodology (equal); supervision (equal); writing – review and editing (equal). **Susan Fuller:** Conceptualization (equal); investigation (equal); methodology (equal); supervision (equal); writing – review and editing (equal). **Andrew M. Baker:** Conceptualization (equal); funding acquisition (lead); investigation (equal); methodology (equal); resources (lead); supervision (lead); writing – review and editing (equal).

## CONFLICT OF INTEREST STATEMENT

The authors declare that there are no competing interests.

## Data Availability

The authors confirm that the data supporting the findings of this study are available within the article [and/or] its supplementary materials. Code for all analysis is available via the Zenodo open access repository: https://zenodo.org/doi/10.5281/zenodo.11189996.

## APPENDIX B

**TABLE A1:** Live (Elliott) trapping effort and trap success for *Sminthopsis douglasi* at Site A in Bladensburg National Park from July 2000 to July 2023.

Date	Trapping effort	Trap success	Source
July 2000	800	3.30%	Mifsud (2000)
April 2001	800	6.75%	Mifsud (2001a)
March 2009	500	0.00	Rich (2009), unpublished
August 2011	537	0.20%	Rich (2011), unpublished
July 2013	1200	0.00	Baker (2013)
October 2013	600	0.00	Baker (2013)
April 2022	100	0.00	This study
May–June 2022	700	0.29%	This study
July 2022	700	1.71%	This study
August 2022	500	0.40%	This study
April 2023	700	0.57%	This study
May–June 2023	700	4.00%	This study
July 2023	700	1.14%	This study

*Note*: All studies used Elliott‐style traps, with traps arrayed at Site A in transect lines comprising a grid of 50–100 traps and each trap spaced 50 m apart. Baits contained a mix of peanut butter and bacon (although Baker, 2013 also compared efficacy of an insect bait comprised of crickets, wood cockroaches, mealworms and plain flour).
